# Different Cranial Ontogeny in Europeans and Southern Africans

**DOI:** 10.1371/journal.pone.0035917

**Published:** 2012-04-27

**Authors:** Marina L. Sardi, Fernando V. Ramírez Rozzi

**Affiliations:** 1 División Antropología, Facultad de Ciencias Naturales y Museo, Universidad Nacional de La Plata - CONICET, La Plata, Buenos Aires, Argentina; 2 UPR 2147 CNRS, Paris, France; Institut de Biologia Evolutiva - Universitat Pompeu Fabra, Spain

## Abstract

Modern human populations differ in developmental processes and in several phenotypic traits. However, the link between ontogenetic variation and human diversification has not been frequently addressed. Here, we analysed craniofacial ontogenies by means of geometric-morphometrics of Europeans and Southern Africans, according to dental and chronological ages. Results suggest that different adult cranial morphologies between Southern Africans and Europeans arise by a combination of processes that involve traits modified during the prenatal life and others that diverge during early postnatal ontogeny. Main craniofacial changes indicate that Europeans differ from Southern Africans by increasing facial developmental rates and extending the attainment of adult size and shape. Since other studies have suggested that native subsaharan populations attain adulthood earlier than Europeans, it is probable that facial ontogeny is linked with other developmental mechanisms that control the timing of maturation in other variables. Southern Africans appear as retaining young features in adulthood. Facial ontogeny in Europeans produces taller and narrower noses, which seems as an adaptation to colder environments. The lack of these morphological traits in Neanderthals, who lived in cold environments, seems a paradox, but it is probably the consequence of a warm-adapted faces together with precocious maturation. When modern *Homo sapiens* migrated into Asia and Europe, colder environments might establish pressures that constrained facial growth and development in order to depart from the warm-adapted morphology. Our results provide some answers about how cranial growth and development occur in two human populations and when developmental shifts take place providing a better adaptation to environmental constraints.

## Introduction

The variation of growth and development among modern humans has been studied since decades. Frequently these studies focused on nutritional and epidemiological aspects that influence life-history variables, such as the age of attainment of adult size, the age at menarche, age at first reproduction, etc., whereas some other studies suggest that differences in growth and development would be genetically programmed [1].

Populations of Sub-Saharan African ancestry, for instance, differ in body size and shape with respect to populations of European ancestry at similar ages and similar socioeconomic levels [1]. The former develop ossification centres and present skeletal maturation and sexual maturation at more advanced ages than the latter; however, these results have been contested [2]. Dental studies also suggest that Southern African populations are characterised by a more advanced development when they are compared with populations of European ancestry [3]–[5]. Comparing adult individuals, craniometric differences were observed in the jaw, midface and cranial base. On average, the upper nasal region is relatively more projected in Europeans, together with more retracted jaws; Southern Africans, in contrast, present low noses in low faces, some degree of prognatism, narrower midfaces and cranial bases and frontal flatness [6]–[9].

Similarities in phenotypes among individuals are produced by regularities in developmental systems but it remains unknown which developmental mechanisms does differ in order to produce variation of specific cranial structures between populations. The study of Strand Vidarsdottir et al. [10] carried on with ontogenetic series dealing with between-populations variation suggests that part of facial shape differentiation is already present in early stages of postnatal ontogeny and that postnatal development contribute to adult differentiation. Even if this study [10] included 10 human groups, some of them were represented by small sample sizes and most of the study focused on the relationship of shape versus size.

All changes produced by growth and development constitute an ontogenetic trajectory. Growth results by changes in size while development by changes in shape [11]–[13] according with biological and/or chronological ages. The parameters that determine an ontogenetic trajectory are: the onset (α) and the offset (β) of growth and development, the rate of change (k) and the initial value of the trait (y_0_), which resulted from growth and development previous to the observation [12].

The link between developmental changes and diversification among species or populations is the heterochronic approach. Heterochrony refers to evolutionary changes in rates and timing of developmental events, which modify ontogenetic trajectories of morphological units. Heterochrony has been described by formalisms of Gould [11] and Alberch et al. [12]. Any modification in α, k and β of a given trait, traditionally measured by a single variable, from one species to other [11]–[13] or from one population to other [14] indicates a heterochronic change. This concept as well as analytical approaches involved underwent several reformulations [13], leading to some confusions.

In the last decades, most of the studies of biological form are based on landmark configuration and shape is quantified by Principal Component Analysis (PCA) after a Procrustes superimposition. Some scholars have suggested that in multivariate comparisons Alberch's et al. terminology [12] cannot be used and some controversy has arisen because there is no consensus about how to interpret ontogenetic trajectories and the dissociation between size, shape and time from multivariate data. On the one hand, Mitteroecker et al. [17], [18] evaluate ontogenetic changes in a shape space between species. These authors state that a change can only be interpreted as heterochrony when their trajectories are identical in the shape space, but differ just in the extension, which indicates that the offset of the development occurs at different time or size. One requisite is that the shape space encompasses all PCs since, according with Mitteroecker et al. [17], [18], individual PCs are statistical constructions and they cannot be directly interpreted. On the other hand, Lieberman et al. [19] consider that an individual PC derived from geometric-morphometric data is an adequate measure of shape because each PC is statistically independent, being useful to derive testable hypotheses about developmental covariation among characters [19]. Lieberman et al. [19] interpret heterochronies from the analysis of single PCs following the method proposed by Alberch's et al. [12] and reinterpreted by Alba [20]. Lieberman et al. [19] state that the requisite for indentifying heterochronies proposed by Mitteroecker et al. [17], [18] is too stringent since the multivariate analysis will almost always result in divergence of one or more PCs, even in two closely related species. Furthermore, the approach of Mitteroecker et al. [17], [18] does not include any measure of ontogenetic time (biological or chronological age), rendering difficult the assessment of heterochronies. Indeed, allometries (size-related shape changes) are sometimes taken as a surrogate of time, but this not always produces similar results because changes in the association between size and shape may be independent of that between shape and age [13], [19]. When age is not available, it has been usual to compare ontogenetic series to explain morphologic divergence, avoiding inferences about heterochronies [21]–[23]. Different approaches can lead to very contradictory interpretations, as occurred in the evaluation of heterochronies between bonobos and chimpanzees. Whereas Mitteroecker et al. [17], [18] explained variation between both species as result of non heterochronic transformations, Lieberman et al. [19], who used biological age as reference for size and shape modifications, suggested that bonobo is paedomorphic relative to chimpanzee due to initial shape underdevelopment.

In this work, we assess craniofacial changes throughout ontogeny in two human populations -Western Europeans and Southern Africans- by means of geometric-morphometric methods. Since we agree with Lieberman's et al. [19], we follow their approach in order to examine main patterns of variation in ontogenetic data. Two null hypotheses are stated: a) Southern Africans and Europeans present similar rates of cranial growth and development, and b) they undergo the offset of growth and development at similar age.

## Results

### Neurocranium

**Figure 1:**
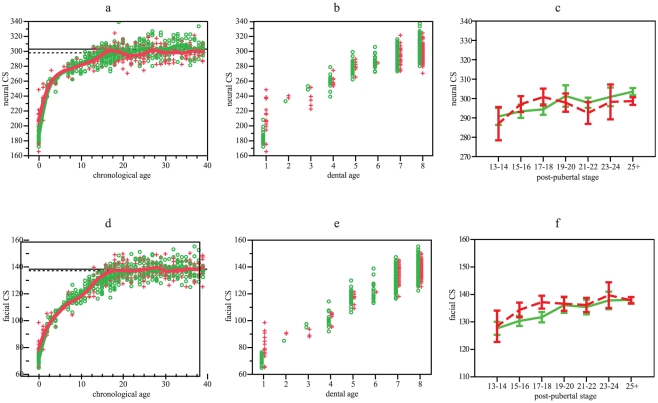
Neurocranial and facial size variation. (a) Neurocranial centroid size values vs chronological age. Smoothing splines accounted for 91.7 and 84.8% of variation for Europeans and Southern-Africans, respectively. (b) Neurocranial centroid size values vs dental age. (c) Mean and 95% standard error of neurocranial centroid size vs post-pubertal stages. (d) Plot of facial centroid size values vs chronological age. Smoothing splines accounted for 92 and 90.4% of variation for Europeans and Southern-Africans, respectively. (e) Facial centroid size values vs dental age. (f) Mean and 95% standard error of facial centroid size vs post-pubertal stages. Green: Europeans. Red: Southern Africans. Horizontal lines in a and b represent adult means: Europeans, solid line; Southern Africans, dotted line.

Size variation can be observed in Figure 1. Neurocranial ontogenetic changes in size were quite similar in both populations showing an important inflection point at ages 3–5 (Fig. 1A). At age 15 most of adult size is attained, however, Europeans achieve greater size (Table 1). According with dental age, changes are gradual being close to adult size around stage 7 (Fig. 1B). Size changes against chronological age (Table 2) on log-transformed data indicate that growth trajectories diverge. Southern Africans present greater size than Europeans at age 0, but it is probably because most of European individuals belong to the first trimester of postnatal life [24]. When individuals of age 0 were removed, slopes do not differ (Table 2). The ANOVA and Dunnet test among post-pubertal stages (Table 3, Fig. 1C) indicate that Europeans show highly significant differences, being those individuals between 13 and 18 years old smaller than adults. Significant difference in Southern Africans is only observed when adults are compared with the class of 13–14 years old. Dental stages 7 and 8 differ in both populations (Table 4).

**Table 1:** ANOVA results for testing differences between adult means.

	F	P
**neurocranium**		
CS	**10.44**	**0.001**
PC1	0.04	0.844
PC2	**5.15**	**0.024**
PC3	**13.86**	**0.000**
**face**		
CS	0.05	0.824
PC1	**298.10**	**0.000**
PC2	**377.90**	**0.000**
PC3	0.56	0.454

**Table 2:** Regression equations and ANCOVA results.

	Regression equations				ANCOVA		ANCOVA without individuals of age 0	
	Europeans		S. Africans		F	F	F	F
neurocranium	constant	slope	constant	slope	intercept	slope	intercept	slope
logCAge vs logCS	**5.29**	**0.13**	**5.38**	**0.10**	**41.14**	**49.68**	**11.20**	0.96
logCAge vs PC1	**0.12**	**−0.04**	**0.13**	**−0.04**	1.25	0.44		
logCAge vs PC2	−0.00	0.00	**−0.01**	**0.01**	0.99	**4.37**	**11.49**	0.18
logCAge vs PC3	**−0.03**	**0.01**	**−0.01**	**0.01**	**45.09**	0.11		
logCS vs PC1	**1.78**	**−0.31**	**2.15**	**−0.38**	**8.12**	**8.08**	0.19	0.72
logCS vs PC2	0.03	−0.01	**−0.31**	**0.05**	**5.54**	**5.84**	**13.53**	0.41
logCS vs PC3	**−0.32**	**0.05**	**−0.40**	**0.07**	**45.39**	0.57		
**face**								
logCAge vs logCS	**4.31**	**0.18**	**4.38**	**0.16**	**21.33**	**17.92**	0.65	2.67
logCAge vs PC1	**−0.12**	**0.05**	**−0.12**	**0.03**	0.03	**49.56**	3.61	**7.31**
logCAge vs PC2	**0.05**	**−0.01**	**0.01**	**−0.01**	**1113.54**	2.27		
logCAge vs PC3	0.00	0.00	**−0.03**	**0.01**	**23.98**	**24.01**	0.38	1.84
logCS vs PC1	**−1.19**	**0.25**	**−0.97**	**0.19**	**17.84**	**25.36**	**9.83**	**13.32**
logCS vs PC2	**0.36**	**−0.07**	**0.44**	**−0.09**	3.34	**9.02**	**1094.94**	1.64
logCS vs PC3	**0.07**	**−0.01**	**−0.28**	**0.05**	**36.80**	**36.50**	0.26	0.10

Numbers in bold indicate probability under 0.05.

**Table 3:** ANOVA and Dunnet's one-tailed test among post-pubertal stages and adults.

neurocranium				
Europeans	CS: F = 10.20	PC1: F = 3.32	PC2: F = 0.55	PC3: F = 5.47
25–39 vs 13–14	p = **0.000**	p = 0.144		p = **0.000**
25–39 vs 15–16	p = **0.000**	p = **0.024**		p = **0.003**
25–39 vs 17–18	p = **0.000**	p = 0.380		p = **0.002**
25–39 vs 19–20	p = 0.612	p = 0.114		p = 0.987
25–39 vs 21–22	p = **0.046**	p = 0.960		p = 0.617
25–39 vs 23–24	p = 0.509	p = 0.999		p = 0.142

S. Africans	CS: F = 2.48	PC1: F = 1.82	PC2: F = 2.17	PC3: F = 1.79
25–39 vs 13–14	p = **0.011**			
25–39 vs 15–16	p = 0.803			
25–39 vs 17–18	p = 0.999			
25–39 vs 19–20	p = 0.888			
25–39 vs 21–22	p = 0.068			
25–39 vs 23–24	p = 0.941			

face				
Europeans	CS: F = 16.93	PC1: F = 3.56	PC2: F = 11.39	PC3: F = 2.46
25–39 vs 13–14	p = **0.000**	p = **0.001**	p = **0.000**	p = **0.003**
25–39 vs 15–16	p = **0.000**	p = 0.054	p = **0.000**	p = 0.123
25–39 vs 17–18	p = **0.000**	p = 0.108	p = **0.000**	p = 0.144
25–39 vs 19–20	p = 0.379	p = 0.205	p = **0.019**	p = 0.338
25–39 vs 21–22	p = 0.165	p = 0.942	p = 0.085	p = 0.505
25–39 vs 23–24	p = 0.929	p = 0.998	p = 0.929	p = 0.373

S. Africans	CS: F = 4.14	PC1: F = 1.74	PC2: F = 0.51	PC3: F = 0.86
25–39 vs 13–14	p = **0.000**			
25–39 vs 15–16	p = 0.073			
25–39 vs 17–18	p = 0.843			
25–39 vs 19–20	p = 0.630			
25–39 vs 21–22	p = 0.543			
25–39 vs 23–24	p = 0.999			

Numbers in bold indicate probability under 0.05.

**Table 4:** ANOVA for dental stages 7 and 8.

	CS	PC1	PC2	PC3
**neurocranium**	F	F	F	F
Europeans	**59.78**	**7.47**	0.70	**8.06**
S. Africans	**10.04**	3.85	0.01	**4.26**
**face**				
Europeans	**76.66**	**10.93**	**75.35**	**5.68**
S. Africans	**19.93**	0.41	3.87	1.40

**Numbers in bold indicate probability under 0.05.**

**Figure 2:**
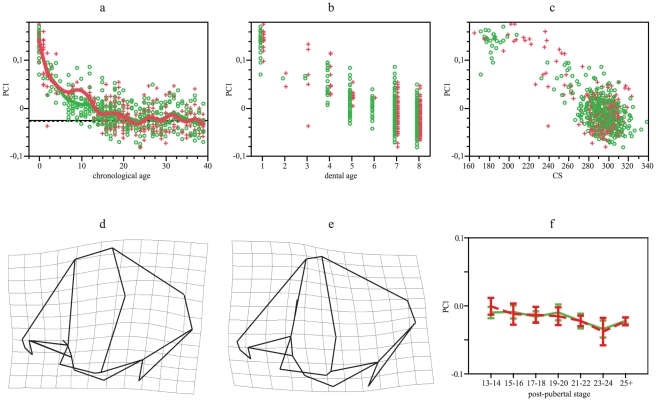
GPA/PCA results for neurocranial PC1. (a) PC1 scores vs chronological age. Smoothing splines accounted for 74% of variation in both distributions. (b) PC1 scores vs dental age. (c) PC1 socres vs CS. (d) Lateral view of neurocranial shape in extreme positive values (newborns = target), considering extreme negative values as the reference. (e) Lateral view of neurocranial shape in extreme negative values (adults = target), considering extreme positive values as the reference. (f) Mean and 95% standard error for PC1 scores vs post-pubertal stages. Green: Europeans. Red: Southern Africans. Horizontal lines in a represent adult means: Europeans, solid line; Southern Africans, dotted line.

From the GPA/PCA for neurocranial landmarks, the first three PCs obtained explain more than 57% of variation. Changes across PC1 (31% of variation) (Fig. 2A–B) show overlapped trajectories for both groups across all ontogeny (the divergence around age 10 may be a consequence of sample bias, see Methods), being adult means non-significant (Table 1). ANCOVA indicate that both trajectories are identical, considering age (Table 2). Allometric trajectories seem quite overlapped (Fig. 2C), but they diverge (Table 2). Nevertheless, since individuals of age 0 may modify slopes, analyses were done extracting them and divergence disappeared (Table 2). Transformation grids (Fig. 2D–E) show an expansion of the neurocranium in lateral view with the relative increment of the anterior (frontal) component as individuals increase in age and size. The ANOVA and Dunnet's test among post-pubertal stages (Table 3, Fig. 2F) indicate that Europeans differ, but only slightly due to the sub-sample of age 15–16; Southern Africans, in contrast, do not differ. Dental stages 7 and 8 differ among Europeans but not among Southern Africans (Table 4).

**Figure 3:**
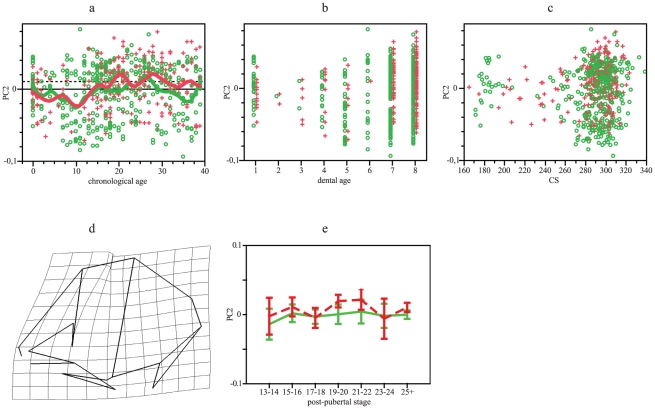
GPA/PCA results for neurocranial PC2. (a) PC2 scores vs chronological age. Smoothing splines accounted for 10.5 and 13% of variation in Europeans and Southern Africans, respectively. (b) PC2 scores vs dental age. (c) PC2 scores vs CS. (d) Lateral view of neurocranial shape, considering extreme negative values as the reference and extreme positive values as the target (other views do not show deformation). (e) Mean and 95% standard error for PC2 scores vs post-pubertal stages. Green: Europeans. Red: Southern Africans. Horizontal lines in a represent adult means: Europeans, solid line; Southern Africans, dotted line.

Between-populations variation across PC2 (14% of variation) according with age and size seems overlapped (Fig. 3A–C), but adults present significant differences (Table 1). According with age, Southern Africans show significant changes, whereas Europeans do not change (Table 2). Trajectories on log-transformed data diverge, but divergence became non-significant removing individuals of age 0. Variation described by PC2 (Fig. 3D) indicates that Southern Africans, as increase in age and size, they develop a less rounded vault with frontal flatness in lateral view. Post-pubertal stages do not differ among Europeans, but they differ among Southern Africans, however, no group is smaller than adults (Table 3, Fig. 3E). Dental stages 7 and 8 do not differ in either population (Table 4).

**Figure 4:**
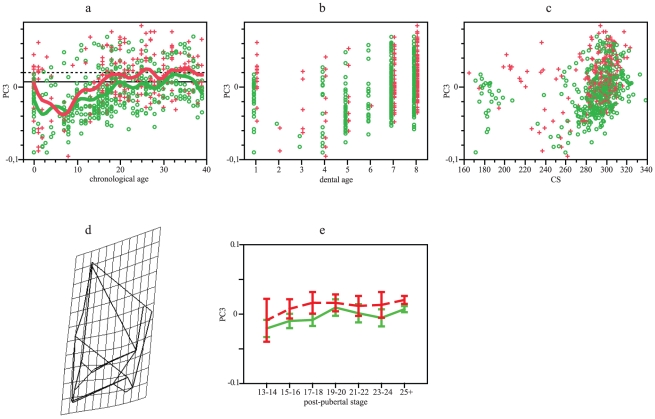
GPA/PCA results for neurocranial PC3. (a) PC3 scores vs chronological age. Smoothing splines accounted for 23 and 19% of variation in Europeans and Southern Africans, respectively. (b) PC3 scores vs dental age. (c) PC3 scores vs CS. (d) Frontal view of neurocranial shape, considering extreme negative values as the reference and extreme positive values as the target. (e) Mean and 95% standard error for PC3 scores vs post-pubertal stages. Green: Europeans. Red: Southern Africans. Horizontal lines in a represent adult means: Europeans, solid line; Southern Africans, dotted line.

According with PC3 (12.5% of variation), Europeans and Southern Africans show similar changes linked to age and size (Fig. 4A–C), showing adults highly significant differences (Table 1). Trajectories are parallel, but intercepts diverge (Table 2). Variation described by PC3 indicates that, since birth, Southern Africans present taller vaults in frontal view (Fig. 4D). In the European sample, individuals under 19 years old differ from adults, but post-pubertal stages do not differ among Southern Africans (Table 3, Fig. 4E). Dental stages 7 and 8 differ in both populations (Table 4).

### Face

Size changes in facial ontogenies do not differ between Europeans and Southern Africans (Fig. 1D–E), neither adult size (Table 1). Slopes diverge as well as intercepts with log-transformed data, however this difference disappears when individuals of age 0 are removed (Table 2). The comparison of post-pubertal stages (Table 3, Fig. 1F) indicated that the offset of facial growth was more advanced in Southern Africans (13–14 years old) than in Europeans (up to 17–18 years old) (Table 3). Variation between dental stages 7 and 8 is highly significant for both populations (Table 4).

**Figure 5:**
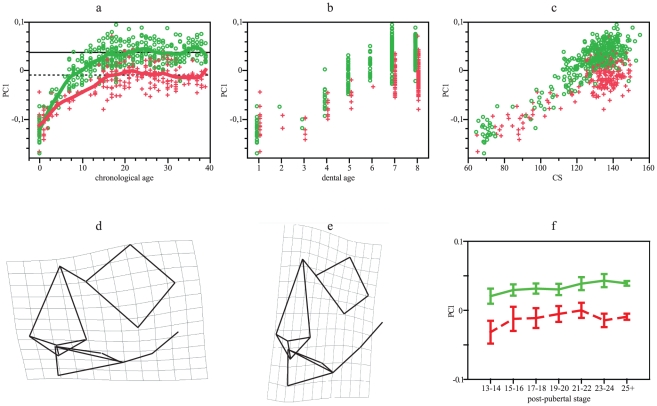
GPA/PCA results for facial PC1. (a) PC1 scores vs chronological age. Smoothing splines accounted for 83.4 and 68% of variation in the European and Southern African distributions, respectively. (b) PC1 scores vs dental age. (c) PC1 scores vs CS. (f) Frontal view of facial shape in extreme negative values (newborns = target), considering extreme positive values as the reference. (g) Frontal view of facial shape in extreme positive values (adults = target), considering extreme negative values as the reference. (g) Mean and 95% standard error for PC1 scores vs post-pubertal stages. Green: Europeans. Red: Southern Africans. Horizontal lines in a represent adult means: Europeans, solid line; Southern Africans, dotted line.

From the GPA/PCA for facial landmarks, the first three PCs obtained explain more than 47% of variation. Trajectories across PC1 (28% of variation), in contrast, show both groups overlapped during the first two years of life, but from this age, trajectories diverge progressively; divergence increases after 5 years old (Fig. 5A) and after dental stage 5 (Fig. 5B), resulting in an important difference in average adult shape among these populations (Table 1). ANCOVA indicates that slopes diverge, but not intercepts (Table 2), which means that there is no important variation among newborns. Adult differentiation is achieved because Southern Africans show lesser shape changes than Europeans. Differentiation between slopes is maintained after removing individuals of age 0 (Table 2). Size-related shape changes on PC1 are also lower in Africans than Europeans (Fig. 5C), being slopes significantly divergent (Table 2). Transformation grids indicate that main postnatal changes occur at the sagital plane; the nasal cavity became taller and narrower, being the nose and the palate more distally located in relation with the orbit (Fig. 5D–E). These changes are more pronounced in Europeans. According with the Dunnet's test, shape modifications are similar in both populations but the non-significant variation of Southern Africans may depend on their greater standard error (Fig. 5F), although differences between dental stages 7 and 8 were significant only for Europeans.

**Figure 6:**
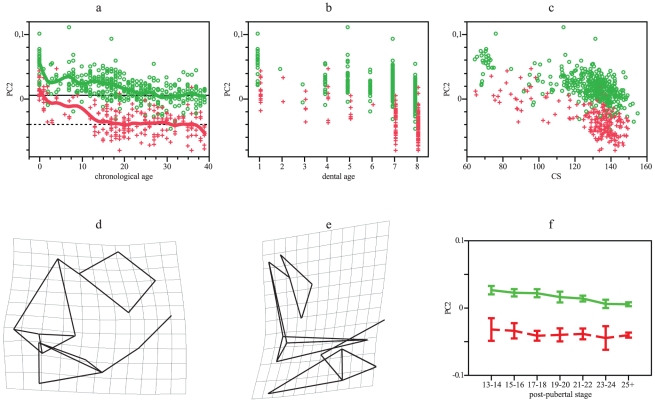
GPA/PCA results for facial PC2. (a) PC2 scores vs chronological age. Smoothing splines accounted for 42 and 46% of variation in the European and Southern African distributions, respectively. (b) PC2 scores vs dental age. (c) PC2 scores vs CS. (d) Frontal view of facial shape in extreme negative values (adult Southern Africans = target), considering extreme positive values (European newborns) as the reference. (e) Lateral view of facial shape in extreme negative values (adult Southern Africans = target), considering extreme positive values (European newborns) as the reference. (g) Mean and 95% standard error for PC2 scores vs post-pubertal stages. Green: Europeans. Red: Southern Africans. Horizontal lines in a represent adult means: Europeans, solid line; Southern Africans, dotted line.

The PC2 (12.5% of variation) expressed variation associated with chronological and dental ages (Fig. 6A–B) and size (Fig. 6C). Adult differentiation is highly significant (Table 1), although both populations show parallel developmental changes since slopes of changes in shape according with log-transformed age and size do not differ (Table 2); the highly significant differences in intercepts reveals that differentiation has begun before birth (Table 2, Fig. 6A–C). Transformation grids indicate that shape changes are related with midfacial morphology (Fig. 6D–E). Southern Africans showed greater prognatism and wider nasal cavity than Europeans during all postnatal ontogeny, but these characteristics are accentuated up to adulthood. Post-pubertal stages do not reveal differences among Southern Africans, neither among latter dental stages, but individuals younger than 19 years old differ from adults among Europeans, as well as individuals of dental stage 7 with respect to those of stage 8 (Tables 3 and 4, Fig. 6D).

**Figure 7:**
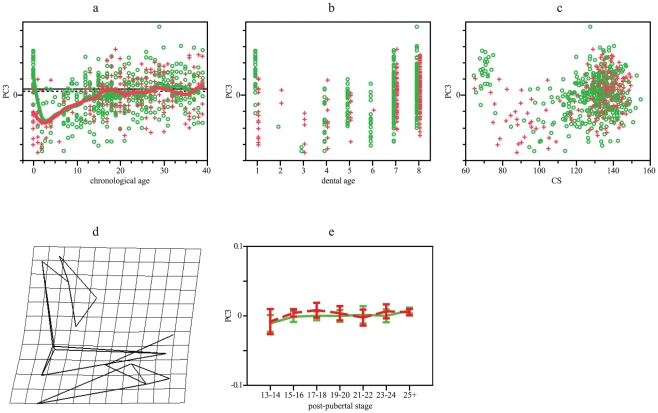
GPA/PCA results for facial PC3. (a) PC3 scores vs chronological age. Smoothing splines accounted for 24% of variation in both distributions. (b) PC3 scores vs dental age. (c) PC3 scores vs CS. (d) Lateral view of facial shape in PC3, considering extreme negative values as the reference and extreme positive values as the target (other views do not show deformation). (e) Mean and 95% standard error for PC3 scores vs post-pubertal stages. Green: Europeans. Red: Southern Africans. Horizontal lines in a represent adult means: Europeans, solid line; Southern Africans, dotted line.

According with PC3 (7% of variation), both populations are quite overlapped across age and size, excepting among the youngest individuals (Fig. 7A–C). Adult shape does not differ (Table 1). Only Southern Africans present significant changes according with age and size (Table 2), diverging from Europeans in slopes and intercepts. Divergence becomes non-significant when individuals of age 0 are removed. Transformation grids indicate that this PC represents ontogenetic increases in prognatism (Fig. 7D). Differentiation among post-pubertal stages and among dental stages 7 and 8 are significant only for Europeans (Tables 3 and 4, Fig. 7E).

## Discussion

Results of this study are somewhat coincident with previous research carried on with adults [6]–[9], regarding neurocranial and facial features, such as nasal morphology, alveolar projection, frontal flatness, among others (Figs. 1, 2, 3, 4, 5, 6 and 7). We demonstrated how those morphologic characters that make people look different are shaped during ontogeny, how some traits covary, and how morphologic traits differ among humans by small scale shifts in developmental rates and timing.

Both the neurocranium and the face express combinations of characters that differ since birth between Europeans and Southern Africans whereas other characters follow postnatal divergent patterns. Diversification produced during postnatal ontogeny is less evident in the neurocranium than in the face because neurocranial growth and development show parallel trajectories for PC1 (Fig. 2). Divergence expressed along other shape components was mainly produced by divergence among individuals belonging to age 0 (Table 2). This is probably because brain growth, which influences on neurocranial size and shape [25], show significant changes in rates during the first year of life [26]. Frontal flatness, a character that distinguishes Southern Africans [8], seems developed after birth (Fig. 3). Increments in the height of the external neurocranial structures, in contrast, are common for both groups and this morphology probably diverge from prenatal ontogeny. In this shape component (PC3), as well as in centroid size, Europeans take more time to achieve adult morphology (Tables 3 and 4; Figs. 1, 4).

Although main postnatal changes are shared between Europeans and Southern Africans, a small proportion of variation, linked to frontal flatness, is divergent due to greater developmental rates in the second group. Thus, both hypotheses proposed are rejected. In heterochronic terms, changes observed in neurocranium have to be interpreted very cautiously. Similar shape at similar age is attained in both populations following PC1, it suggests that there is no heterochrony between populations. In PC2, Europeans do not show change through age whereas Southern Africans do; PC2 represents most probably changes that cannot be interpreted from heterochrony. Differently, PC3 indicates a displacement, aspects represented by this PC are observed in Southern Africans at an earlier age that they occur in Europeans.

Facial ontogenetic trajectories are more divergent between populations (Tables 2, 3 and 4; Figs. 5, 6 and 7). The main axe of shape variation does not show differences at birth but progressively diverges, showing Europeans higher developmental rates than Southern Africans (Fig. 5) and attaining taller faces. During adulthood, Europeans attain highly significant shape differences with respect to Southern Africans, but with similar size (Table 1).

Other facial traits, linked to nasal width and prognatism, show parallel ontogenetic changes and are already different at birth (Fig. 6). Size-related variation is divergent because allometries are more pronounced in Southern Africans (Table 2). These ones present all over postnatal ontogeny wider noses and the superior alveolar arch more projected than Europeans. This differentiation may result from prenatal divergence and accentuated during postnatal ontogeny, along with changes in nasal height (Fig. 5).

Facial size and those morphologic characters represented by main PCs attain their adult state during pre-pubertal stages in Southern Africans and later in Europeans (Tables 3 and 4). Even if the smaller sample size among the former may produce statistical type II error for shape components, when dental age was used Southern Africans do not change after the second permanent molar is at the occlusal plane, contrarily to what occurs among Europeans. This indicates that Southern Africans undergo the offset of facial growth and development earlier than Europeans (Tables 3 and 4).

Considering facial ontogeny, thus, both hypotheses were rejected. Overall differentiation between both populations arises by a combination of processes that involve changes in rates and time of offset of facial growth and development. Facial development involves increases in facial and nasal height, being more accentuated in Europeans than in Southern Africans. These ones seem to retain younger traits during adulthood. In heterochronic terms, face in Europeans followed acceleration [20]; it undergoes greater changes in shape, with respect to age and size, with a similar final size (Table 2, Fig. 2). This set of characters does not differ at birth but progressively diverge.

Chronological age in Southern Africans might be biased by aging methods (see Methods). However, the use of a biological age as dental eruption produced similar results to those obtained with chronological age (Fig. 2). Dental maturation is supposed to be more advanced in Southern Africans [3]–[5], thus dental categories may encompass African individuals that are younger than Europeans, all of which expresses that ontogenetic differences would be more pronounced than observed here.

Although morphologic variation is continuous, significant differences for some body and cranial characters have been observed among worldwide modern human populations, especially for those that are geographically distant [6]–[9]. The pattern of variation has been explained mainly as a consequence of population history. Environmental factors seem to have a minor influence in overall morphology [27], [28]; however, some characters are likely to differ, as a consequence of selection or plasticity [29]–[33].

Lower limbs, whose morphology expresses climatic adaptation, also differ among these same populations, being Southern Africans taller than Europeans. The ontogenetic study of Frelat and Mitteroecker [34] indicates that this pattern results from postnatal divergence (in lower limb length) and prenatal divergence (relative length of tibia and femur). However, whereas our results of facial morphology suggest greater developmental rates for Europeans than Southern Africans, developmental rates of femur and tibia are greater among the latter. Postnatal ontogeny would reinforce the body climatic adaptation [34].

Similarly, nasal variation has been regarded as environmentally shaped. Nasal morphology varies across ecogeographic regions probably as a consequence of climatic adaptation [29], [31], . Native populations of colder climates present taller and narrower noses with respect to warm-adapted populations (e.g. Subsaharan Africans), providing greater surface for warming and humidifying inspired air through the contact with the nasal mucosa, which enables a better thermoregulation [29], [31], [32]. Since this morphology is established at birth and accentuated early in postnatal life, it may express adaptative pressures involved.

These results may present evolutionary implications, providing clues for Neanderthal characters. Neanderthals have been considered as hominids adapted to cold climate, in part due to their supposed large paranasal sinuses. Recently, Rae et al. [35] demonstrated that Neanderthals were not characterized by relatively large paranasal sinuses, neither are they relatively smaller, as would be expected according with experimental studies of cold adaptation [33]. Neanderthals present relatively wide nasal apertures -associated to prognatism-, which is a character related to warm climate [36]. This paradox was explained by Holton and Franciscus [36], who suggested that a relative wide nasal aperture in Neanderthals is the retention of a plesiomorphic character. Facial morphology of Neanderthals differ from modern humans since very early in ontogeny [37], [38]. The similar pattern of midfacial variation in Neanderthals and Southern Africans may suggest that facial ontogenies were also similar, along with more advanced maturation in the former [37], [39] as well as the latter [1], [3]–[5]. At the end of the Pleistocene, when modern *Homo sapiens* migrated into Asia and Europe, colder environments established pressures that might constrain facial growth and development for subsequently attain adapted morphology. This morphology was attained by increasing facial developmental rates and extending the attainment of adult size and shape but without affecting the reproductive output of the population.

The offset of cranial growth and development, especially in facial structures, differs between Europeans and Southern Africans, which clearly fits with developmental timing in other traits [1], [3]–[5]. It is probable that genes and several substances that act on development are involved. Some circulating hormones, such as growth hormone and IGF I, have effects on overall growth, promoting also growth on particular tissues or stimulating the local production of other growth factors [40]. Growth hormone and IGFI regulate systemically developmental rates and times of maturation, as is evident during the adolescent growth spurt. They may influence on ontogenetic allometries –e.g. the face- together with –or as a result of- other major developmental events, which occur quite late in ontogeny, and also probably associated to other variables of life-history, such as sexual maturation. Unfortunately, to the moment, there is no actual evidence suggesting differences in growth-promoting substances between native sub-Saharan populations and Europeans.

In sum, this study highlights the importance of examining the intra-specific variation in phenotypes and development for understanding evolutionary origins of interspecific diversification [41]. The adult differentiation between Europeans and Southern Africans arises by a combination of processes that involve traits modified during prenatal life and also others that diverge during postnatal ontogeny. If evolutionary developmental paleoanthropology is better defined by the questions it asks: how, when and why [42], our results provide some answers about how cranial growth and development occur in two human populations and when developmental shifts take place across individual's life. This enables to infer why variation does occurred, probably as a by product of the integration with other biological variables providing a better adaptation to environmental constraints. A further concern is that when anatomically modern humans are compared with other hominids, inferences about the differentiation must explicitly consider which human population is being compared.

## Methods

Two cranial ontogenetic series derived from individuals whose age at death is between 0–39 years old were studied (Table 5). The West European sample encompasses, for the main part, Portuguese cemetery-derived individuals, which are housed at the Museo Antropologico, of the University of Coimbra (Portugal). A smaller proportion of this sample is composed of cadaver-derived skulls from French individuals, which are housed at Musée de L'Homme (France). Sex and age at death is known through cemeteries archives and direct observation of cadavers in the case of sex.

**Table 5:** Sample distribution according with chronological ages.

	Europeans				Southern Africans		
chron. age	females	males	unknown	Total	females	males	Total
***0***	1	1	24	26	3	8	11
***1***	0	0	1	1	6	4	10
***2***	2	0	5	7	2	2	4
***3***	1	1	0	2	0	2	2
***4***	0	0	6	6	2	1	3
***5***	0	0	1	1	0	0	0
***6***	0	1	0	1	0	1	1
***7***	6	2	2	10	1	2	3
***8***	5	5	2	12	0	1	1
***9***	2	2	1	5	0	1	1
***10***	6	3	2	11	0	0	0
***11***	4	4	0	8	0	0	0
***12***	9	3	1	13	3	0	3
***13***	2	5	0	7	2	4	6
***14***	5	2	4	11	2	1	3
***15***	8	10	2	20	1	4	5
***16***	6	7	0	13	2	8	10
***17***	14	8	1	23	1	8	9
***18***	11	9	1	21	2	9	11
***19***	6	5	0	11	6	2	8
***20***	3	6	0	9	6	8	14
***21***	5	4	0	9	4	7	11
***22***	8	3	0	11	2	2	4
***23***	2	6	0	8	2	1	3
***24***	6	5	0	11	2	3	5
***25–39***	60	65	0	125	47	46	93
**Total**	**172**	**157**	**53**	**382**	**96**	**125**	**221**

The second sample encompasses South African individuals of Bantu origins. The cranial material belongs to the Dart collection housed at the University of Witwatersrand (Johannesburg, South Africa). This collection comprises skulls of cadaver-derived origins. Sex was assessed by direct observation, whereas age at death was estimated by unknown methods [43].

Since chronological age may be biased in the Dart collection, dental maturation was recorded according with a ranking (Table 6) in both collections, which is a good proxy of biological development. Each dental class was established when some teeth are fully emerged. Thus, morphometric analyses were carried out considering as reference both chronological and biological (dental) ages.

**Table 6:** Ranking of dental maturation and sample distribution.

		Europeans				Southern Africans		
stage	maturation	females	males	unknown	total	females	males	total
**1**	no teeth at the occlusal plane	1	1	24	26	5	10	15
**2**	dec. incisors at the occlusal plane	0	0	1	1	1	1	2
**3**	dec. canines at the occlusal plane	0	0	2	2	4	1	5
**4**	dec. dentition completely erupted	3	2	10	15	3	6	9
**5**	1st permanent molar at the occlusal plane	16	12	6	34	3	4	7
**6**	3rd premolar is at the occlusal plane	9	8	2	19	1	0	1
**7**	2nd permanent molar at the occlusal plane	77	60	8	145	25	24	49
**8**	3rd molar fully erupted	66	74	0	140	54	79	133
	**Total**	**172**	**157**	**53**	**382**	**96**	**125**	**221**

Thirty three-dimensional (3D) landmarks, located in the vault, basicranium, and face (Table 7) were registered with Microscribe on the left side of the skull by one of the authors (M.L.S.). Wire-frames were built with landmarks located either on the face or neurocranium (Table 7).

**Table 7:** Landmarks registered with Microscribe on the left side of the skull.

neurocranium	face
Nasion	Nasion
Glabella	Subspinale
Bregma	Prosthion
Vertex	Palatine-maxillare suture
Lambda	Posterior nasal spine
Opisthocranion	Right alare
Opisthion	Left alare
Basion	Zygomaxillare
Hormion	Maximum alveolar width
Pterion	Maxillary tuberosity
Eurion	Inferior zygo-temporal suture
Asterion	Dacryon
Porion	Ectoconchion
Stephanion	Superior rim of the orbit
Posterior mandibular fossa	Orbital
Sphenotemporal crest	
Optic foramen	
Dacryon	
Superior rim of the orbit	

All 3D coordinates of landmarks were analysed by geometric-morphometric methods. Geometric-morphometrics suit well with the analysis and representation of the relationships among size and shape because it enables the evaluation of heterochronies since the Procrustes superimposition provides measures of shape once all information due to scale, location and rotation was removed; and it provides a measure of size –the centroid size- that is uncorrelated with shape.

Cuadratic distances between homologous landmarks were minimised by means of Generalised Procrustes Analysis (GPA) with *Morphologika*. After Procrustes transformation, landmark configurations were analysed by means of a Principal Component Analysis (PCA). GPA and PCA enabled to obtain scores of shape variation and the centroid size (CS). Transformation grids were built to visualize morphologic changes.

Neurocranium and face were analysed separately because both morphological units present different embryological origins and they present different developmental rates [44], [45]. The neurocranium encompasses two main skeletal structures –the vault and the basicranium- of different embryological origins, however growth rates are similar and associated to brain growth. From a phylogenetic perspective, some developmental shifts in both the neurocranium and the face can explain morphologic diversity among mammals [46] and, more specifically, among primates [19], [47].

Individuals were plotted according with size (CS) and shape (PCs) variables against chronological and dental ages. In order to visualize trajectories according with chronological age, the smoothing spline was adjusted with Jump 5 (SAS Institute Inc.). This method requires the definition of the smoothing parameter λ, which establishes the trade-off between the bias and the variance along a trajectory. Some λ were explored but 10 were chosen by visual inspection. Greater detail is provided in those PCs that account for greatest percentage of variance.

Statistical analyses were done with an alpha level of 0.05 using with Systat 10.2 (Systat Software Inc.) and Statistica (Statsoft Inc.) softwares. Differences among adults were tested with ANOVA. In order to test for change of size against age, shape against age and shape against size, within-populations regression lines were adjusted after the transformation of chronological age and centroid size into natural logarithms to get linear distributions. Equality in trajectories was evaluated by means of ANCOVA. Firstly, ANCOVA for testing the homogeneity of slopes was performed introducing the interaction term between the covariate and the grouping variable. Population was the grouping variable, log-chronological age and log-centroid size were covariates and shape variables (PCs) were the dependent variables; log-centroid size was also a dependent variable using log-chronological age as a covariate. A non significant interaction between the grouping variable and the covariate indicates that the relation between the covariate and the response variable Y does not differ between groups. When slopes did not differ, a second ANCOVA pooling the regression slopes (removing the interaction term) was performed. This enabled to test for differences in y-adjusted values for any x-value, which is also a test or equality of populations intercepts [48]. When slopes and intercepts do not differ, trajectories are identical and potential adult variation may result from the extension or truncation of trajectories; but if intercepts differ, it is probably due to differentiation generated during prenatal life. A significant interaction, in contrast, indicates that slopes differ. When slopes differ significantly, certain values of X (i.e. age) were chosen and both ANCOVA methods were repeated with and without the interaction term in order to determine the regions of significance [48]. Significant slopes can be associated with non-significant intercepts, which may indicate that both groups are not different during first stages of postnatal ontogeny and they diverge later. If the intercept also differs, no assumption about ontogeny can be done because the differentiation between intercepts is not maintained for other values of X [48].

In order to evaluate the offset of growth and development, individuals of different chronological ages in each population were compared with ANOVA and Dunnett's test (one tail). The Dunnett's t test is a method for comparing several group means to a control mean, which is useful to look for significant differences of those individuals that are older than 12 years old with respect to the adult reference. Adults encompass individuals aged between 25 and 39. Those individuals aged from 13 to 24 were grouped into 13–14, 15–16, 17–18, 19–20, 21–22, 23–24 classes in order to get greater sample sizes. When dental age was used, adults are those individuals belonging to dental class 8. These ones were compared only with those of dental class 7 which approximately corresponds to post-pubertal stage, given that M2 is fully emerged around 12.5–13.5 years [49]. Differences between means were compared with ANOVA.
